# The Effect of Ingredient Item Depiction on the Packaging Frontal View on Pre- and Post-Consumption Product Evaluations

**DOI:** 10.3390/foods8080354

**Published:** 2019-08-20

**Authors:** Nicole Timmerman, Betina Piqueras-Fiszman

**Affiliations:** Marketing and Consumer Behaviour Group, Social Sciences, Wageningen University and Research, Hollandseweg 1, 6706 KN Wageningen, The Netherlands

**Keywords:** perceived deception, mismatch perception, expected/perceived flavor intensity, packaging cues, rational cognitive style, experiential cognitive style

## Abstract

The current research focused on the (in)congruity between pictorial (ingredient item depiction) and textual (ingredient list) information on food packaging, namely, an apple–mango juice. Specifically, the influence of these information sources on expected and perceived flavor intensities, mismatched perceptions, perceived deception, and intention to purchase was studied by taking into account the possible moderating role of consumers’ thinking style. Three studies were performed, the first and third at a Dutch University by means of surveys and sensory tests, and the second via an online survey. The results showed that, overall, most consumers did not perceive the incongruity between pictorial and textual information as mismatching. However, a perceived mismatch from packaging, whether originated by the design manipulations or not, did increase perceived deception and lowered willingness to purchase. This effect was robust for both mismatches, among packaging elements (pre-consumption) and from expected and perceived flavor ratios (post-consumption), but was more substantial for the post-consumption mismatch. Although the moderating effect of cognitive processing style regarding expected and perceived flavor ratios from pictorial and textual (ingredient list) information was not confirmed, the results indicated that the effect of salient textual information is substantial, independent of a particular processing style or label usage.

## 1. Introduction

The food industry is continuously growing. Consumers, therefore, face an ever-increasing number of daily products to choose from in supermarkets. In this abundant choice environment, consumers have a limited capacity to process all the information they face when deciding on their food choices and usually rely on effortless and intuitive thoughts. Consequently, a product’s pictorial appearance is often the main driver for consumers’ first purchase decisions [1,2].

One of the strategies for pictorial or pictorial design elements of a packaging often used by companies is providing information about the product within [3]. Depiction of ingredient item images on the front of a packaging is one of the most frequently used cues explicitly linked to the content of the product and is usually prominently positioned on the front-of-pack (FOP) label [4]. These images allow consumers to create expectations and draw inferences about the product, its quality, and its taste [3,5,6,7].

However, these tempting ingredient images on the FOP label do not always correspond with the actual content of the product. Hence, there are two situations in which a potential mismatch between the FOP information and the actual content can occur. First, in pre-consumption evaluations, comparing the depicted ingredient elements on the FOP label with the actual ingredient list can lead to contrast perceptions based on the formed expectations from the ingredient images if this greatly differs. Second, when a consumer relied on the ingredient item depiction to buy the product, a post-consumption mismatch between the expected and perceived flavor can arise at home after tasting the product. In both situations, it can be questioned whether ingredient item depiction is a misleading factor on the packaging and whether it leads to negative consequences.

Pictorial and verbal stimuli are both part of the product packaging elements. Benefits of using one over the other to communicate a product’s message have been researched previously in the domain of advertising [8,9,10,11]. Research addressing this distinction in packaging cues has shown a contrast in the way of processing the information from pictorial and verbal sources in terms of processing style and cognitive load [12]. To process verbal cues, a higher level of cognitive load is needed in comparison to pictorial cues, as pictorial cues require more unintentional and unconscious processing, evoking a higher vividness effect [13,14]. Also, pictorial stimuli tend to draw consumers’ attention in store at the point of purchase [15,16], which contributes to a quick evaluation allowing consumers to form expectations and inferences more easily compared to reading text [14]. In other words, pictorial cues, such as depiction of ingredient items, may lead to a faster inference-making process based on existing knowledge, previous experiences, and associations [17].

Nonetheless, the impact of verbal cues, also known as textual cues, on information transmission should not be underestimated [18]. The effect of certain textual cues (e.g., product names and nutritional content) on product packaging design have increasingly gained attention in research over the last decade [19,20], and have been found to explain a large part of product expectation formation [21,22,23]. However, in comparison to textual stimuli, consumers tend to rely more on pictorial stimuli in considering purchase decisions at the point of sale [24].

### 1.1. Literature Background on Consumer Product Evaluations from Pictorial Design Elements in Food Labelling

Images are usually prominently positioned on the FOP label [4]. Recent studies have focused on the effect of image depiction of the whole product on the packaging of food products on expectation formation (e.g., [25]) and on willingness to buy (e.g., [26]). Madzharov and Block [27] demonstrated that pictorializing more product units on the FOP label can increase actual consumption. Similar results were found by Neyens, Aerts, and Smits [28], as consumption increased as a result of a larger depicted image of the product on the packaging. Deliza, MacFie, and Hedderly [29] found that adding pictures on juice packaging significantly altered expected sensory attributes. This shows that consumers transferred their previous experiences with the pictures to the product expectation of the drink. In addition, Rebollar et al. [25] recently showed that sensory expectations were altered according to the way in which crisps were presented on the packaging, and that this accordingly changed willingness to buy. In comparing ready-to-eat crisps to raw potatoes on the packaging, consumers expected the actual crisps to be crunchier and saltier and ratings of willingness to purchase were higher in the former case.

As opposed to this growing domain of research on the image depiction of a product as a whole on the FOP label on consumers’ product expectations, limited research has been done on the impact of depicting ingredient items on the FOP label as a means to communicate actual content on consumers’ expectations. Moreover, even fewer studies have addressed consumers’ corresponding actual flavor perception by showing such (in)congruent images with the actual content on the FOP label. The first and only study known by researchers studying the effect of (in)congruency between the product content and the depiction of ingredient items on the FOP label on actual flavor perception was published in Japanese by Sakai and Morikawa [30]; the study was summarized in English by Mizutani et al. [31]. They analyzed the influence on hedonic and sensory evaluations either showing congruent (e.g., depicting an orange on the FOP label when drinking orange juice) or incongruent (e.g., depicting an apple on the FOP label when drinking orange juice) combinations. Scores on sensory attributes and palatability were found to be higher with congruent combinations. More recently, Machiels and Karnal [18] conducted a study in which participants had to drink a glass of orange juice, while simultaneously being exposed to its commercial packaging. In a 2 × 2 between-subjects design, stimuli on image depiction (either a whole orange or a glass of orange juice) and textual cues (processed versus unprocessed) were manipulated to measure taste evaluations and willingness to buy. Their results showed that depicting the juice image led to purer taste evaluations and that, for certain consumers, showing an image of an orange increased purity of taste and willingness to buy.

This empirical research shows that pictorial cues can, indeed, alter product evaluations to a large extent, yet the explaining theories and underlying mechanisms are lacking. Some studies have focused on expectation formation in the pre-consumption phase, while others have shown effects in altering actual flavor perceptions in the post-consumption phase. This paper will address effects of ingredient item depiction in both phases, to cover the entire product experience.

### 1.2. Explaining Theories

The cues on a packaging design are used by consumers to predict the benefits of a certain product, based on personal beliefs and associations. This benefit extraction from product cues is called the cue utilization process [32,33,34] and is explained by two phases. First, a consumer must perceive a cue to be able to predict a certain benefit from it. Hence, only cues that are sufficiently salient will be noticed and perceived by a consumer [32,35]. Another term used for this phase is the belief formation process, as presented stimuli are perceived by the consumer using their cognitive structure. The second phase follows naturally in transforming the cue perceptions into inferences. The notion of these inferences lays in the predictive value of the cue perception towards a product benefit [35]. Differently stated, the cue utilization process describes the extent to which perceived packaging cues are used to predict sensory pleasure, which in its place is linked to flavor intensity evaluation of the product.

In previous research, congruency (no mismatch) among different cues on a product’s packaging design has shown to positively influence product evaluations such as brand impressions, perceived product value, and willingness to purchase [36,37,38]. Congruency is defined as a certain extent of conformity between stimuli, cues, and features of the presented product [39]. 

Moreover, in food products effects of congruency between pictorial and actual food content have shown to positively affect hedonic evaluations. The previously mentioned Japanese study from Sakai and Morikawa [30] showed that apple juice tasted better and flavors were perceived to be more intense accompanied with a pictorial of an apple compared to pictorials of an orange or the control (no pictorial). From these findings, research suggests that incongruence amongst different product cues, in contrast to congruence, elicits more negative product evaluations. Negative consumer responses after a perceived deviation from expectations, regardless of the direction of this deviation, can be explained by the basic model of *generalized negativity* proposed by Carlsmith and Aronson [40]. This model proposes that whenever a discrepancy is perceived between expectations and the actual product experience, a hedonically negative state in the individual is generated. However, a more sophisticated model, the “*assimilation-contrast model*” [41,42], explains that whenever moderate incongruity occurs, incongruence between inferred expectations and actual perception, the percept could assimilate to what is expected. When the difference between inferred expectations and actual perception is large enough, contrast (i.e., mismatch perception) might occur instead.

In the case of food packaging, after having generated product expectations based on the extrinsic product attributes in the pre-consumption phase, the consumer will compare these product expectations with the actual product experience after tasting in the post-consumption phase. The brain will try to avoid discrepancies between what was expected and what was experienced [42]. Discrepancy in this case could be the difference in perceived flavor/ingredient ratio between what was expected and what was experienced. When the discrepancy among these two is relatively small, assimilation between the expectation and the experience will be likely to occur. The evaluation of the product will then shift into the direction of the expectation and the product perception will become similar or equal to the product expectation. However, when the discrepancy between expectation and experience is too big, contrast (i.e., mismatch) will occur [29,43,44]. As a result, the consumer will magnify the mismatch and, consequently, the product perception will become (very) different from the product expectation [45], with potential negative consequences as a result. 

#### 1.2.1. Perceived Mismatch and Perceived Deception

The literature on deception essentially states that deception is viewed as an act that misleads the target party [46], in this case, the consumer. In the domain of marketing, Gardner [47] developed a product and consumer-based definition, describing the concept more behaviorally oriented and adding a dimension of perception. In his definition, Gardner [47] states that deception occurs whenever a marketing element leaves a consumer with an impression or belief deviating from what could have been known with proper knowledge and that this impression or belief is factually untrue or potentially misleading. Moreover, he used the term *perceived deception* as the feeling of being fooled or tricked by marketing. In this study, incongruence amongst packaging elements could enhance perceived deception. 

Whenever stimuli on a packaging are incongruent and with this communicate an ambiguous message towards the consumer, no accurate or clear inferences about the product can be made. In addition to creating confusion in assessing the identity of a product, incongruent stimuli might also lead to perceived deception. For instance, a product that claims to be “lowered in sugar” but still has 50 percent of sugar per 100 g, may lead consumers to confusion in assessing the healthiness of the product and the product’s benefits, but also might leave the consumer with a feeling of being fooled or tricked. Following the statement that fluent information processing in general leads to more positive product evaluations [48] and taking into account that stimuli congruence in product appearance has earlier shown to stimulate credibility evaluations [49], congruence expressed across ingredient item depiction on the packaging and the actual presented ingredient item list (no mismatch in pre- and post-consumption evaluations) was expected to lower the perceived deception.

In other words, while depicting images on a product’s packaging serves several positive benefits, being potentially dishonest in overemphasizing product pictorials should be taken into account, as this might affect consumers’ perceived deception and corresponding willingness to (re)purchase. 

#### 1.2.2. Purchase Intention Following Perceived Deception 

In this research, it was proposed that perceived deception leads to lower intentions to purchase the product. Research has shown that perceived deception leads to lower ratings of satisfaction [50,51]. Moreover, it is known that satisfaction with a certain product increases the attitude towards that product [51]. A consumer’s attitude towards a product is, in turn, considered as the most important predictor of the behavioral intentions of consumers [52]. 

In addition, satisfaction has shown to have a direct effect on the trustworthiness of the product by the consumer [37,53]. However, whenever stimuli on the product’s packaging are communicating inconsistent information, this is likely to cast doubt on a product’s functioning (i.e., incongruence negatively affects credibility) and, with this, questioning of the trustworthiness of the product [53]. Since trustworthiness of a product can be seen as a mediator for intentions to purchase the product, higher ratings of perceived deception are expected to lower a consumer’s intention to purchase the product. 

### 1.3. Individual Differences in Cognitive Processing Style

Within decision-making research, two broad basic preferences are distinguished: intuitive and deliberative decision-making, which is rooted in several dual process theories (e.g., [54,55,56,57,58]). Most widely acknowledged in dual processing theory on decision making is Kahneman’s System 1 and System 2. System 1 can be described as the more implicit, intuitive, automatic, effortless, associative, and fast system. In contrast, system 2 is characterized by reasoning in which processing goes slower, takes more effort, and happens more conscious. A general assumption in dual processing theory is that individuals differ in the degree they use intuition and deliberation in perception, thinking, and solving problems. 

A study by Ares, Mawad, Giménez, and Maiche [59] is the first and only study known by the authors which evaluated the effect of measured rational or intuitive cognitive style on information processing and consumer choices based on FOP labels. By means of an eye-tracking experiment, differences between rational and experiential processers could be distinguished in terms of their reliability on various elements of the label. A distinction between rational and intuitive thinkers was found in reliability on complex information, such as nutritional information or on graphic design to make inferences about the product and base decisions on, respectively. 

Based on the abovementioned literature and the preliminary findings about the influence of thinking style on consumer evaluations of food products, it seems reasonable to expect that individual differences will moderate the reliability on pictorial or verbal packaging cues in expected fruit ratio and perceived mismatch between the actual and depicted ingredient items on the packaging.

### 1.4. Hypotheses

**Figure 1:**
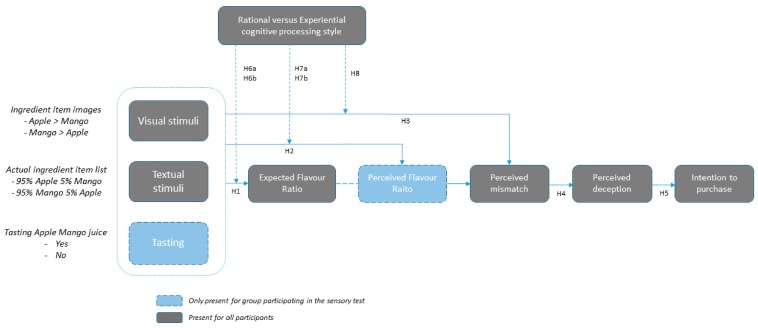
Conceptual model and corresponding operationalization of levels to the factors.

The aim of this research was to increase understanding of the effect of depicting ingredient items on the front of packaging on pre- and post-consumption product evaluations related to flavor expectation and perception, perceived deception, and purchase intention, as well as the role of consumers’ cognitive processing style. Three studies were conducted to test the following hypotheses (see the conceptual model in Figure 1):

**Hypothesis** **1.** Regardless of the ingredient list shown, expected flavor ratio will be assimilated to the depicted ingredient item images on the front of packaging.

**Hypothesis** **2.** Regardless of the ingredient list shown and the flavor of the juice, perceived flavor ratio will be assimilated to the depicted ingredient item images on the front of packaging.

**Hypothesis** **3.** In both the pre- and post-consumption evaluation, (in)congruency between the pictorial and textual packaging elements will lead to (mis)match perceptions.

**Hypothesis** **4.** A perceived mismatch in both the pre- and post-consumption phases will increase ratings of perceived deception.

**Hypothesis** **5.** Perceived deception will negatively affect an individual’s intention to purchase the product.

**Hypothesis** **6.** Before tasting the juice, (a) consumers classified as dominant experiential processors will rely more on pictorial stimuli of the packaging in their expectation formation compared to textual stimuli and, therefore, assimilate their expected flavor ratio towards the depicted ingredient item images; and (b) consumers classified as dominant rational processors will rely more on textual stimuli of the packaging in their expectation formation compared to pictorial stimuli and, therefore, base their expected flavor ratio on the shown ingredient item list. The opposite pattern of results is expected from dominant rational processors.

**Hypothesis** **7.** After tasting the juice, (a) consumers classified as dominant experiential processors will rely more on pictorial stimuli compared to textual stimuli and will, therefore, assimilate their perceived flavor ratio towards the depicted ingredient item images; and (b) consumers classified as dominant rational processors will rely more on textual stimuli compared to pictorial stimuli and will, therefore, assimilate their perceived flavor ratio towards the ingredient item list.

**Hypothesis** **8.** In both the pre- and post-consumption phases, consumers classified as dominant rational processors are significantly more likely to consciously perceive a (mis)match between pictorial ingredient item depiction on the FOP and the textual ingredient item list, compared to dominant experiential processors.

## 2. Study 1

### 2.1. Participants

At least 40 Dutch students per condition irrespective of age and gender was aimed for, resulting in a minimum response of 320 respondents finishing the questionnaire, of which at least 160 participated in the sensory test (*post-consumption evaluation*). Respondents were recruited via convenience sampling at the Wageningen University by randomly approaching people at the university, posting a message onto different social media pages, via flyers, and by means of the researchers’ personal networks. The test took place in a computer room at Wageningen University. The only requirements were that participants liked 100% fruit juice in general and had to be of Dutch nationality, to limit familiarity with the product stimuli. This factor was controlled for during the gathering of participants. Participants were also asked for any allergies before starting the sensory testing and received a chocolate incentive after participation.

### 2.2. Image Stimuli

A “100% fruit juice” was chosen as the research stimulus because it is a product often depicting ingredient items on the FOP label. In addition, a wide variety of 100% fruit juices are available in Dutch supermarkets in terms of both flavors and brands. 

**Figure 2:**
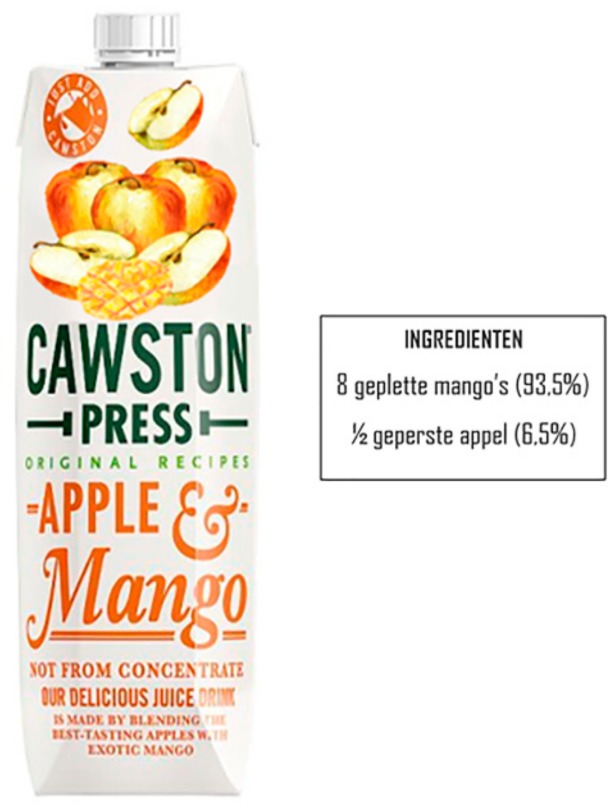
Example of the condition pictorializing more apples incongruent with the ingredient list.

Four 100% fruit juice labels were designed from existing packaging in which only two factors were modified; the ingredient item depiction on the FOP label (pictorial) and the actual ingredient list containing the percentages per ingredient of the juice (textual). Two levels were considered in the depiction of ingredient items, with on the one hand, a *high-expensive ratio* for the expensive ingredient (mango) compared to the cheap ingredient (apple) and, on the other hand, a *low-expensive ratio* of these fruits. Combining these levels to create the stimuli for this research resulted in two congruent combinations and two incongruent combinations. A pictorial representation of one of the four conditions can be seen in Figure 2. 

All other elements of the packaging; shape, color, typology, weight, size, product title, and brand were kept identical amongst the designs. The stimuli were adapted from the “Apple & Mango” juice packaging of the British brand “Cawston” using Adobe Photoshop software. As the study took place in the Netherlands where Cawston juice was not retailed at the time of research, recognition bias should have been minimal, and participants could not base their evaluations on prior experiences with the brand. A pre-test amongst Dutch students (*N* = 16), of which 10 were female, was performed to ensure an appropriate measurement tool regarding noticeability of the pictorials on the packaging. Also, all manipulated stimuli were checked on “realistic looks” by the same students.

### 2.3. Design

In this study, the effect of ingredient item depiction in the pre- and post-consumption phase was investigated by means of a 2 × 2 × 2 between-subjects experiment. The influence of three manipulated variables was tested (see Table 1): *pictorial ingredient elements* in terms of ingredient item depiction (Apple > Mango ratio versus Apple < Mango ratio); *textual ingredient elements* in terms of actual ingredient list (Apple > Mango ratio versus Apple < Mango ratio); and a tasting test (half of them tasted a juice sample, and the other half did not).

### 2.4. Procedure

After entering the room, participants were seated behind a computer screen. Desks were separated by screens so that participants could not see each other or the packages that other participants received. Participants were told to first read the introduction and informed consent on the screen. After agreeing to the informed consent, participants were presented with one of the four images of a 100% juice package. First, they were asked to answer three general statements about the package in order for them to pay attention to this package (“I like the design of this package”, “The packaging of this 100% fruit juice looks attractive to me”, “The text on the front of the package is easy to read”) all on a seven-point Likert scale ranging from completely disagree to completely agree. None of these questions specifically focused on the ingredients of the juice.

On the next page, the image of the juice was no longer visible, and respondents were asked to answer several questions about this packaging. The first page contained a question concerning the expected intensity of the flavor(s) including two distractors; mango, apple, banana, and orange “Please rate the expected intensity of the following ingredients” on a slider from 0 to 100. 

Participants in the tasting conditions received a sample (40 mL) of the juice, after which the survey continued identically to the non-tasting conditions. The sampled juice (a premium brand of not-from-concentrate apple–mango juice) was kept identical in each condition and was served within half an hour after pouring from fridge temperature (4 °C). The juice contained the same two ingredients as the manipulated stimuli (apple 93.5% and mango 6.5% juice). A blind pre-test (*N* = 11) showed that participants were unable to clearly identify mango or apple from the juice. 

Next, perceived deception was measured by two items adapted from Darke and Richie [60] on a seven-point Likert scale (α = 0.88) (“I feel tricked by the packaging of the 100% Apple Mango juice”, “I feel betrayed by the packaging of the 100% Apple Mango juice”). In order to measure intention to purchase, items from Grewal et al. [61] and Dodds et al. [62] were adapted (α = 0.84) (“It is very likely that I would consider this product”, “I would definitely intend to buy this product”, “If I were going to buy a 100% apple–mango juice in the future, there would be a high probability of choosing this product”).

On the next page, a specific question on the perceived mismatch between the stimuli presented with was asked for (“Did you perceive a mismatch among the shown stimuli?”). Whenever this question was answered with yes, the respondent was automatically guided to a question to check to what degree this mismatch was perceived between the depicted ingredient images and the actual ingredient item list (“The mismatch I perceived was completely determined by the combination of the depicted ingredient item images and the ingredient list next to the packaging”). For the groups that tasted a juice, the degree to which this mismatch was caused by a discrepancy between their expected and perceived flavor ratio was checked (“The mismatch I perceived was completely determined by the flavor intensity”). If the response to the first question was negative, these questions were automatically skipped.

For those in the tasting conditions, the juice sample was provided again and respondents were asked to rate their perceived intensity of the flavors banana, apple, and mango (“Please rate how strong you perceive the intensity of the following ingredients”) on a slider from 0 to 100. Moreover, participants were asked for their frequency of buying 100% fruit juices in general by choosing from “daily”, “weekly”, “monthly”, “yearly”, or “never”. Also, they could indicate the degree to which they like 100% fruit juices in general and the specific combination of fruit they were showed with, by checking one of the boxes on a seven-point scale. In addition, two control questions were asked in order to check whether participants were familiar with the brand Cawston and if they ever consumed a juice from the brand before. 

Following this, all respondents were asked questions about their dominant cognitive processing style, in order to test for possible moderating factors of individual processing style on expected/perceived flavor ratio and (mis)match perceptions regarding the packaging elements. In order to determine a respondent’s dominant cognitive processing style, items from the revised version of the Rational-Experiential Inventory (REI) scale developed by Pacini and Epstein [63] were used. Originally, the revised scale (REI-40) had a separate scale for rational and experiential thinking styles, corresponding to analytic and heuristic processing, respectively. The two subscales consist of *need for cognition* (NFC) (α =0.90) and *faith in intuition* (FI) (α = 0.82). Respective to NFC and FI, both scales consist of 20 items, divided by another subscale of engagement and ability, all measured on a 5 point scale ranging from 1 (*completely false*) to 5 (*completely true*). Reliable shortened versions of the REI-40 have been used previously (e.g., [64]). In this study, the shortened version of the REI was also used, consisting of the 5 highest scoring items in the factor analysis on NFC and the 5 highest scoring items in the factor analysis on FI. To minimize the order effect, the statements were presented in a randomized order. A previously created Dutch translation of the REI, developed by Witteman, van den Bercken, Claes, and Godoy [65], was used, as this study was aimed at the Dutch consumer. Lastly, the participants were asked to provide their demographic information. This included questions regarding gender, age, and current study status.

### 2.5. Data Analysis

In this study, the independent variables were *pictorial ingredient item depiction, textual ingredient list,* and *tasting.* The hypotheses concerned the effect of these variables on product evaluations, which were measured with the help of five constructs: *expected/perceived flavor ratio*, (*mis*)*match perceptions*, *perceived deception*, and *intention to purchase.* Statistical analysis of the main and interaction effects of the packaging elements on these constructs were measured by means of the test of equal proportions and factorial ANOVAs, reporting the effect size for both pre- and post-consumption evaluations of the packages. 

Furthermore, the influence of mismatch perception on perceived deception was tested with a one-way ANOVA and the influence of perceived deception on intention to repurchase was measured by linear regression analysis.

#### Moderation Effect

In order to check for possible moderating effects of cognitive processing style, the REI questionnaire had to be analyzed to verify that the scale, indeed, measured the two constructs of interest (i.e., rational and experiential thinking). First, responses on items were reversed if these were negatively framed in the survey. 

A maximum likelihood analysis with orthogonal (Varimax) rotation was conducted on the 10 items of the REI with a Kaiser–Meyer–Olkin sampling adequacy of 0.78. The two factors clearly showed eigenvalue’s greater than 1 and together explained 37.75% of the variance of which 23.15% accounted for the factor “Intuitive” (α = 0.796) and “Rational” (α = 0.647), respectively. The items clustering on the same components were indeed similar to the division between the NFC and FI scales of the REI. To split participants as either being more rational or more intuitive processors, the distance from the median was calculated on each scale for each participant. This so-called Processing Style Influence (PSI) score was developed with the following equation: PSI score = ((Median Rationality Score) − (Actual Rationality Score)) + ((Actual Experientiality Score) − (Median Experientiality Score)). A negative PSI score reflects a dominant R-processor and a positive PSI-score places a participant in the dominant E-processor category. These PSI-categories were used to test the hypotheses on dominant thinking style [66]. If a significant effect of PSI category was found, PSI scores were considered to check the degree of difference. Factorial ANOVA and a test of equal proportions analysis were performed to check for the possible moderating effect of cognitive processing style on expected flavor ratio and (mis)match perceptions, respectively

All data were analyzed via the statistical software package IBM SPSS Statistics 22.0 using a significance level of *p* ≤ 0.5. To prevent multicollinearity, all predictors were rescaled using effect coding [67,68]. Pictorial, textual, tasting, and PSI-category were centered around the means before computing the interaction terms, which were together with the main effects entered into the models. When a significant difference between the conditions was detected, the strength of the effect of the factor on the affected dependent variable was of interest. 

### 2.6. Results

A total of 475 respondents participated in the study, with 229 in the tasting conditions (post-consumption evaluation) and 246 in the non-tasting (pre-consumption evaluation). Respondents who were familiar with the brand “Cawston” and those who responded in less than four minutes (*N* = 30) were removed from the sample. The final sample consisted of 436 Dutch students (M = 20.9, SD = 2.3) of which 214 were in the pre-consumption conditions (104 males) and 222 in the post-consumption conditions (108 males) and were taken into account for data analysis. 

A one-way ANOVA to check the randomization of participants for the control variables showed that participants were equally distributed amongst the different conditions (Table 2). A chi-square test of independence (*N* = 436) showed that both gender (*X*^2^(1) = 3.99, *p* = 0.780) as well as PSI category (*X*^2^(7) = 2.98, *p* = 0.887) were also equally distributed amongst the eight conditions.

#### 2.6.1. The Effect of Pictorial and Textual Packaging Elements on Expected Flavor Ratio

Table 3 provides an overview of the results in every condition. The first step in the analysis was to check whether the independent variables affected the expected flavor ratio measured on a subtraction scale expected for apple–mango (*N* = 436).

Results showed a main effect of the ingredient list shown next to the package (textual) on the expected flavor ratio with a large effect size (*F*(1432) = 280.05, *p* < 0.05, *ω*^2^ = 0.394), indicating a higher expected mango flavor compared to apple when showing a higher percentage of mango in the ingredient list (M = −46.7, SD = 2.6), compared to showing a higher percentage of apple on the ingredient list (M = 15.4, SD = 2.7) on an individual’s expected flavor ratio.

No main effects of the ingredient item depiction (pictorial) on the expected flavor ratio (*F*(1432) = 1.82, *p* = 0.178) and the interaction effect of pictorial and textual ingredient item information (*F*(1432) = 1.58, *p* = 0.209) were found. 

In other words, and contrasting the hypothesis, regardless of the depicted ingredient image on the front of the package, consumers assimilated their expected flavor ratio towards the ingredient item list shown.

#### 2.6.2. The Effect of Pictorial and Textual Packaging Elements on Perceived Flavor Ratio

Opposite to expectations, again no main effect was found for the ingredient item depiction (pictorial) on the perceived flavor ratio (*F*(1218) = 0.26, *p* = n.s.; Table 3). A main effect was found for the ingredient list shown next to the package (textual) on the perceived flavor ratio (*F*(1218) = 4.89, *p <* 0.05, *ω*^2^ = 0.017). This indicates that individuals’ perceived flavor ratio was also assimilated towards the textual packaging cue.

**Figure 3:**
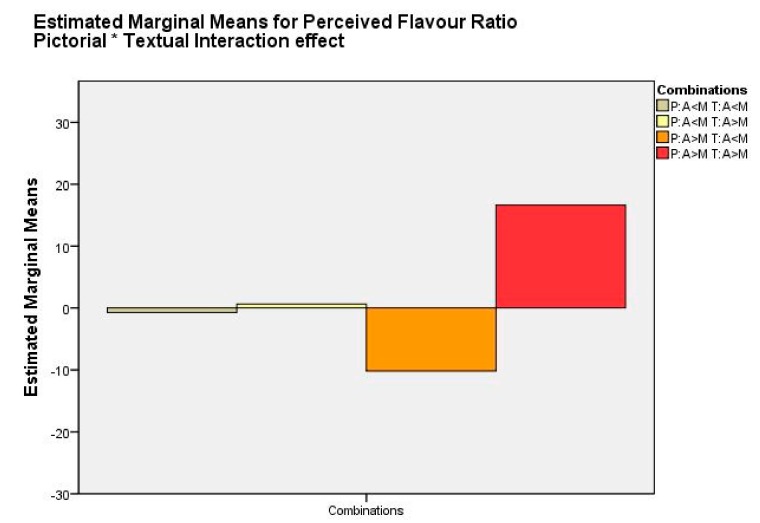
Perceived flavor ratio of different FOP images for the different ingredient lists shown. Note: P stands for pictorial, T stands for textual, A < M stands for dominant mango, A > M stands for dominant apple.

Furthermore, the interaction effect between pictorial and textual ingredient items on the perceived flavor ratio showed a significant result with a small effect size (*F*(1218) = 3.98, *p* < 0.05, *ω*^2^ = 0.013). This indicates that the different images were affected differently accompanied by the different ingredient lists (see Figure 3). If the picture with more apples compared to mangos was shown, dependent on the ingredient list shown next to it, participants either perceived a dominant mango flavor (M = −10.2, SD = 6.4) or a dominant apple flavor (M = 16.6, SD = 6.3), indicating assimilation towards the ingredient list shown whenever more apples were depicted on the front of packaging. 

However, the figure shows that for the pictorial with more mango compared to apple, the perceived flavor ratio for apple and mango only marginally differs, in both cases being nearly zero, indicating a 50:50 flavor ratio regardless of the ingredient list shown next to it. Meaning that the apple percept was diminished by the label. In other words, and contrasting the hypothesis, textual packaging cues are a stronger predictor of perceived flavor ratio compared to pictorial packaging information, but mostly when more apples (versus more mangoes) are displayed.

#### 2.6.3. The Effect of (in)Congruency Amongst Pictorial and Textual Packaging Elements on (mis)Match Perceptions

A chi-square test of equal proportions was conducted in order to check whether mismatch perceptions differed between both the congruent and incongruent conditions in the pre-consumption phase (Table 4). A significant test result (X^2^(1) = 3.99, *p* = 0.038) showed that mismatch perceptions differed amongst congruent and incongruent conditions in the pre-consumption phase. In line with the proposition, in the congruent conditions, 16.0% of respondents perceived a mismatch, which is substantially lower compared to 27.8% in the incongruent conditions. 

#### Pre-Consumption Evaluation

Subsequently, to check for which combinations of pictorial and textual information this was the case for, another cross tabulation was created with perceived mismatch set out against the four conditions of which two were congruent and two were incongruent (Table 5). Chi-square test of independence again showed a significant result (*X*^2^(1) = 1.289, *p* = 0.001). Unexpectedly, but in line with the interaction effect in Figure 3, in the incongruent condition, visualizing more mango on the picture accompanied with an ingredient list indicating a large amount of apple, the mismatch was not clearly perceived. Only in the incongruent condition depicting more apples on the front of pack accompanied with an ingredient list showing more mangos, 41.8% of the respondents perceived this incongruity as a mismatch.

In the congruent conditions, no mismatch between the ingredient image on the front of packaging and the ingredient list on the bottom of the pack was present. Therefore, additionally, the answers of the 17 respondents indicating a mismatch in the congruent conditions were further analyzed. Out of 17 perceived mismatches in the congruent conditions, 15 were completely unrelated to the ingredient information on the packaging (e.g., “I think a lot of sugar is added to these kind of drinks”, “I think a transparent packaging would suit 100% juice better”, “I did not taste so I do not know”). The other two did indicate a mismatch between ingredient item depiction and the ingredient list, of which one respondent commented on the name of the juice being “Apple Mango” juice, suggesting more apple while (in their condition) mango was the main ingredient which was considered mismatching.

#### Post-Consumption Evaluation

A chi-square test of equal proportions was conducted in order to check whether mismatch perceptions differed between the congruent and incongruent conditions in the after tasting of the product in the post-consumption evaluation (Table 4). In contrast to the non-tasting group, for the tasting group in the post-consumption evaluation, a non-significant test result (X^2^(1) = 0.334, *p* = 0.563) showed that mismatch perceptions did not differ amongst congruent and incongruent conditions. Perhaps tasting the juice overpowered the visual recall of the image on the packaging. As the results for mismatch perception between congruent and incongruent conditions was inconsistent in the pre-consumption evaluation and non-significant in the post-consumption evaluation, no clear conclusions can be drawn on the effect on incongruity between pictorial and textual packaging elements on mismatch perceptions.

#### 2.6.4. Influence of Mismatch Perception on Perceived Deception

In line with expectations, a significant effect was found between mismatch perceptions and perceived deception (*F*(1434) = 178.98, *p* ≤ 0.05), indicating that people who perceived a mismatch (M = 4.1, SD = 1.4) felt more deceived by the packaging of the juice compared to people who did not perceive a mismatch (M = 2.9, SD = 1.2). Hypothesis 4 can, therefore, be accepted. 

Additionally, to check whether mismatch perceptions serve as a mediator between packaging information and perceived deception, a full factorial ANOVA was run with IVs *pictorial, textual,* and *tasting* and their two- and three-way interactions on the DV *perceived deception.* No significant main effects were found from the image shown on the package (*F*(1428) = 1.14, *p* = 0.286), the ingredient list shown next to the package (*F*(1428) = 1.78, *p* = 0.182), or from tasting the product (*F*(1428) = 0.25, *p* = n.s.) on perceived deception. Furthermore, none of the interaction effects among these variables were found to be significant either, with *pictorial × textual* (*F*(1428) = 1.41, *p* = n.s.), *pictorial* × *tasting* (*F*(1428) = 2.13, *p* = 0.145), *textual* × *tasting* (*F*(1428) = 0.17, *p* = n.s.), and *pictorial* × *textual* × *tasting* (*F*(1428) = 3.59, *p* = 0.059). In other words, neither the packaging information nor tasting the product had a direct effect on perceived deception. However, from the strong significant effect of the ANOVA from perceived mismatch on perceived deception, as described previously, a mediating role of mismatch perceptions on perceived deception was evident. Therefore, it can be concluded that perceived deception is mediated by perceived mismatch.

Additionally, and to see which of the two possible mismatches plays a larger role in feelings of perceived deception, two linear regressions were performed on the scores among the people who did perceive a mismatch (*N*_total_ = 117). In the non-tasting group (*N =* 47), the regression only contained mismatch perceptions between pictorial and textual ingredient information and amongst the perceived mismatches in the tasting conditions (*N* = 70) for both mismatch perceptions; between *pictorial and textual ingredient information* and between the *expected and perceived flavor intensities* were included in the model.

For the mismatch perceptions in the non-tasting group, a significant regression equation was found for mismatch perceptions between the depicted ingredient items and the actual ingredient list in the non-tasting group (*F*(145) = 17.27, *p* < 0.05), with an *R*^2^ of 0.190. The linear regression showed that mismatch perceptions between the depicted ingredient items and the actual ingredient list, indeed, affected perceived deception, with an increase of perceived deception with 0.21 points out of 7 for each point increase in perceived mismatch. 

For mismatch perceptions in the tasting group, again a significant regression equation was found (*F*(267) = 8.36, *p* < 0.05), with an *R*^2^ of 0.200. Here, the mismatch from packaging elements was found to be non-significant (*p* = 0.062). However, in this model, only the mismatch between expected and perceived flavor ratio was significant (*p* < 0.05) with a constant of 1.840 and a B of 0.402, indicating that an increase in mismatch perception between the expected and perceived flavor ratio of one unit, increased perceived deception by 0.4 point out of seven, which was much larger compared to the increase in perceived deception from the mismatch in the non-tasting group. These results suggest that a mismatch between the expected and perceived flavor ratio plays a larger role on perceived deception than the mismatch in pictorial and textual packaging elements.

No difference of perceived deception was found among respondents who saw the congruent (non-misleading) ingredient information pack and the incongruent (misleading) ingredient information pack (*N* = 436), though results indicated a non-significant trending in the predicted direction indicating higher ratings of perceived deception for the incongruent condition (M = 2.7, SD = 1.4) compared to the congruent condition (M = 2.9, SD = 1.5), (*t*(434) = −1.17, *p* = 0.243).

#### 2.6.5. Influence of Perceived Deception on Willingness to Purchase

It was confirmed that greater feelings of perceived deception led to a lower intention to purchase the product (*F*(1434) = 50.35, *p* = 0.001), with an *R*^2^ of 0.104. Participants’ predicted willingness to purchase the juice decreased with 0.29 point out of 7 whenever perceived deception increased with one point measured on a seven-point scale. In other words, and in line with the proposition, the more deceived a person feels from the product, the lower their willingness to purchase it will be.

Additionally, an independent-samples *t*-test was conducted to compare willingness to purchase between respondents who saw the congruent (non-misleading) ingredient information pack and the incongruent (misleading) ingredient information pack (*N* = 436). Results indicated a non-significant trend in the predicted direction indicating higher willingness to purchase for the congruent condition (M = 4.7, SD = 1.3) compared to the incongruent condition (M = 4.6, SD = 1.3), *t*(434) = 0.43, *p* = 0.668.

#### 2.6.6. Processing Style Influence as a Moderating Factor

Hypotheses 6–8 considered the moderating effect of dominant cognitive processing style on expected and perceived flavor ratio and perceived mismatch. None of the analyses revealed significant effects (Table 6), suggesting that the effect of the pictorial and textual elements on these variables did not depend on whether individuals were experiential (E) or rational (R) processors.

#### Interim Discussion

**Figure 4:**
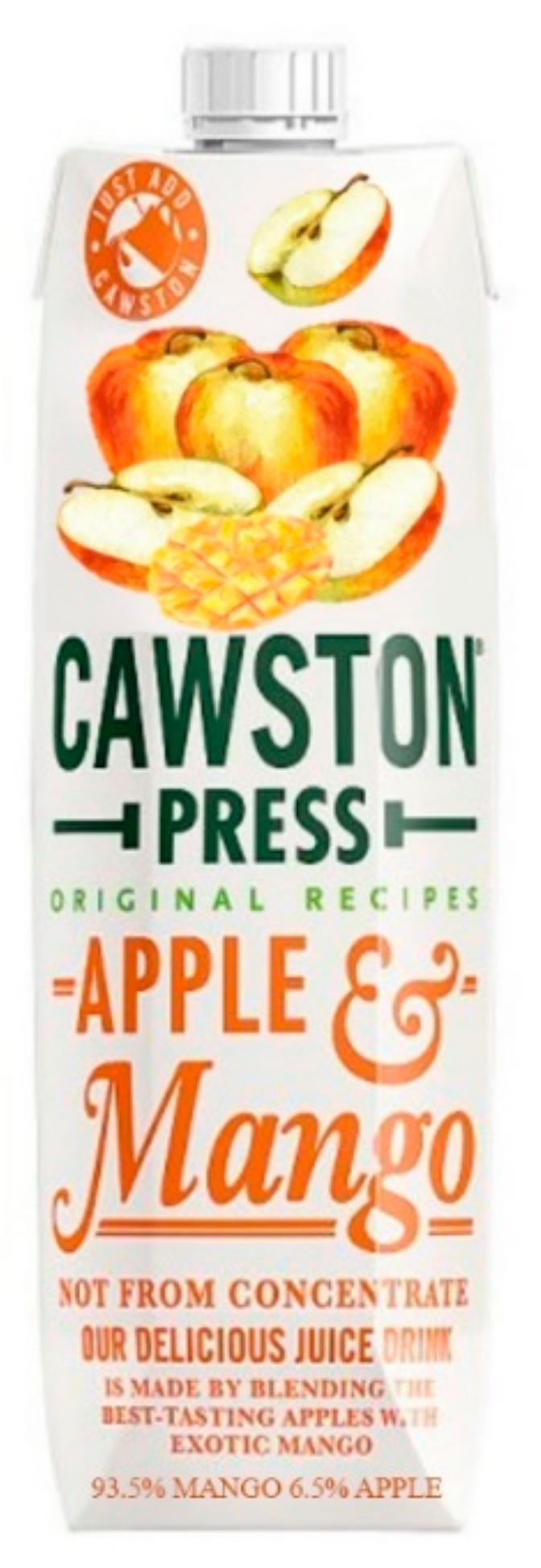
Example of one of the combinations of the packaging including the ingredient list as used in Study 2.

A conceptual replication of the first study was conducted to check whether the deviating findings from the first study could be due to the cue salience of the textual information. In the first study, the manipulation showed the packaging including a pictorial of the ingredient items, accompanied with the corresponding textual information in a box next to the packaging. The salience of the textual information might have drawn most attention. Therefore, the first study was replicated for the pre-consumption evaluation online (Study 2), with a new design of the stimuli (see Figure 4 for an example). In this manipulation, the textual information was made less salient on the package to create a more realistic design. Furthermore, the analyses performed were identical to those in the first study.

## 3. Study 2

### 3.1. Participants

The recruitment and screening criteria were the same as in the first study. The final sample consisted of 216 Dutch students (57 males) (M = 22.8, SD = 4.9) who were further considered for data analysis (Table 7). All scales used to measure the constructs had an adequate internal consistency; willingness to purchase (α = 0.87), and perceived deception (α = 0.82). Factor analysis with varimax rotation verified the two factors of the REI again: intuitive (α = 0.76) and rational (α = 0.68). 

### 3.2. Results

#### 3.2.1. The Effect of Pictorial and Textual Packaging Elements on Expected Flavor Ratio

Table 8 provides an overview of the results for the outcome on different dependent variables in every condition.

Similar to the first study, a main effect of textual package element on the expected flavor ratio with a large effect size was found (*F*(1214) = 27.40, *p* < 0.05, *ω*^2^ = 0.141). 

In line with previous expectations and in contrast to the first study, a main effect of pictorial package information was found (*F*(1214) = 5.58, *p* < 0.05, *ω*^2^ = 0.018). With both visuals, consumers expected the juice to be dominant in mango flavor, with a mean difference of 13.1 (compared to a non-significant mean difference of 5.1 in the first study). No interaction effect of pictorial and textual ingredient item information on expected flavor ratio was found (*F*(1214) = 1.68, *p* = 0.197).

#### 3.2.2. The Effect of (in)Congruency Amongst Pictorial and Textual Packaging Elements on (mis)Match Perceptions

A perceived mismatch from (in)congruent information was measured (*N* = 216) between the congruent and incongruent conditions (Table 9). Again, mismatch perceptions did not differ amongst congruent and incongruent conditions in this second study (*X*^2^(1) = 0.026, *p* = 0.873). 

Additionally, the answers of the 25 respondents indicating a mismatch in the congruent conditions were further analyzed. Out of 25 perceived mismatches in the congruent conditions, 18 were completely unrelated to the ingredient information on the packaging. The other seven did indicate a mismatch between ingredient item depiction and the ingredient list, of which two respondents commented on the name of the juice being “Apple Mango” juice, suggesting more apple while (in their condition) mango was the main ingredient which was considered mismatching. 

#### 3.2.3. Influence of Mismatch Perception on Perceived Deception

Similar to the first study and in line with expectations, a significant effect was found between mismatch perceptions and perceived deception (*F*(1214) = 42.30, *p* < 0.05), indicating that people who perceived a mismatch (M = 4.1, SD = 0.2) felt more deceived by the packaging of the juice compared to people who did not perceive a mismatch (M = 2.7, SD = 0.1). 

Additionally, an independent-samples *t*-test was conducted to compare perceived deception between respondents who saw the congruent (non-misleading) ingredient information pack and the incongruent (misleading) ingredient information pack. Results indicated a trend in the predicted direction indicating higher ratings of perceived deception for the incongruent condition (M = 3.2, SD = 1.4) compared to the congruent condition (M = 2.9, SD = 1.5); however, this result was non-significant (*t*(214) = −1.81, *p* = 0.072).

#### 3.2.4. Influence of Perceived Deception on Willingness to Purchase

Similar to the first study and in line with expectations, the more deceived a person feels from the packaging, the lower this person’s intention to purchase (*F*(1214) = 32.36, *p* < 0.05), *R*^2^ = 0.127. Participants’ predicted willingness to purchase the juice was equal to 4.916–0.362 (perceived deception level) when perceived deception was measured on a seven-point scale. 

Additionally, no difference again was found between willingness to purchase in the congruent (M = 3.9, SD = 1.4), compared to the incongruent (M = 3.8, SD = 1.4) conditions (*t*(214) = −0.586, *p* = 0.559).

#### 3.2.5. Processing Style Influence as a Moderating Factor

Again, no moderating effect of dominant processing style on the effect of packaging information on expected flavor ratio (*F*(1208) = 0.01, *p* = 0.980) and mismatch perceptions (Table 10) was observed. 

#### Interim Discussion

In combination of the two studies, it can be said that regardless of an individual’s processing style, a less salient positioning of textual information leads to an effect on the ingredient image. In both studies, respondents were asked to focus on the packaging before filling in the questionnaire. However, it is evident that in real-life situations of evaluating food packaging, consumers have other things on their mind when grocery shopping [69]. In this context, consumers are less likely to look at the small letters on the back of packaging such as factual ingredient information [70,71,72]. Another study was performed aiming to create a more realistic design, in which the questions to focus on the pack were removed and instead consumers had to remember an 8 digit number to increase their cognitive load [73].

**Figure 5:**
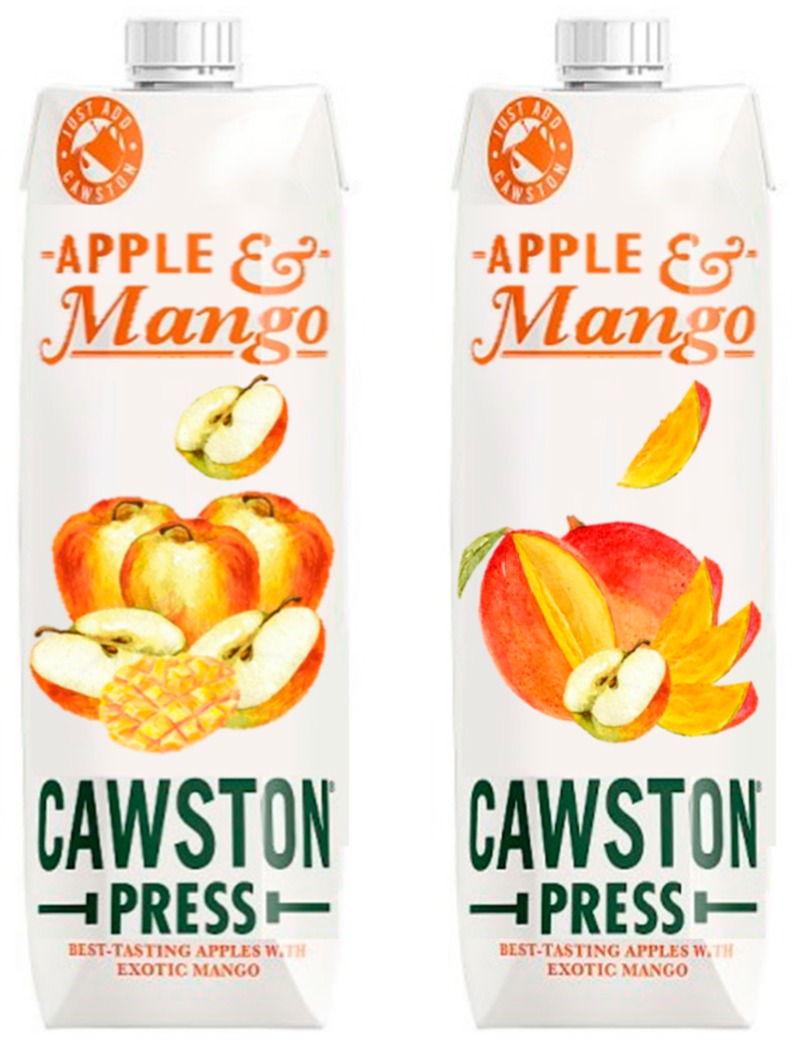
Manipulations of the packaging as used in Study 3.

A new design of the stimuli was created and pre-tested with a more salient positioning of the image and the extra text on the package was deleted, moreover no textual ingredient information was available (see Figure 5) resulting in two conditions only. Also, an additional measure of taste (hedonic ratings) was included in the previous survey. The main goal of adding the element of “tasting” to the research was to investigate differences in perceived flavor ratios and how a mismatch between these and expected flavor ratios would influence levels of perceived deception and willingness to purchase. Liking of the product’s taste could make consumers disregard feelings of deception induced by disconfirmation of expectations and, therefore, was added.

Lastly, the REI inventory was used to determine a consumer’s dominant processing style. A pitfall of using the REI for this study was that being either a more rational or more experiential processor is context specific [64]. Therefore, these ten items were replaced by items on image and ingredient label use as a possible moderator in expected and perceived flavor ratio from images [74]. Furthermore, the analyses performed were the same as in the first study.

## 4. Study 3

### 4.1. Participants

A total of 114 respondents participated in the tasting test. All of the participants tasted the juice this time, the only between-subject factor was the design of the packaging (see Figure 5). The same screening criteria as in the previous studies was applied. The final sample consisted of 108 Dutch students (49 males; M = 20.9, SD = 1.8) that were taken into account for data analysis. 

### 4.2. Procedure

Data were collected in a computer room at Wageningen University. Participants were asked to fill in a questionnaire and taste a 100% fruit juice. The sensory test was a replicate from the first study with a few alterations described next. To create a more realistic setting, participants were given a higher cognitive load to gain more spontaneous answers [73], respondents were shown an 8 digit number (53209695) that they were asked to remember by memory and needed to recall later in the survey. The researcher did not allow participants to write down the number. After answering the question on perceived flavor intensities, respondents were asked to fill in the 8 digit number. In order to check for the moderating effect of label usage, participants had to answer the question taken from a study performed by the “Consumentenbond” in the Netherlands in 2017 on information on food packaging, “*how often do you look at the following elements*” rated never to always on a 5 point scale for the following list: *brand, imagery, ingredient list, country of origin, product name.* To check whether these were indicators for image usage and ingredient list usage, three items from the same questionnaire were adapted and added as well: “*the image on the front of packaging gives an honest impression of the content*”, “*the ingredient prominently shown on the front of packaging gives the impression that the product contains a lot of it*”, and “*the image on the front of packaging is purely decorative*”. Also, two items were taken from the Australian “Consumer Label Survey 2015” and translated to Dutch: “*I usually look at the ingredient list when I buy a product for the first time”* and “*for me personally, the information on the ingredient list is important when I buy a product for the first time”*. 

An extra control question was added to check whether participants liked the juice they tasted which might influence their intention to purchase regardless of a perceived mismatch from packaging. Moreover, a question on a study program with the options *nutrition and health, food technology* or *other* was added. 

Finally, the items highlighting the focus on the packaging were taken out to stimulate more spontaneous answering of the questions. The REI scales were also removed from this study provided the null effects obtained above.

### 4.3. Data Preparation and Sample Description

All scales used to measure the constructs had an adequate internal consistency: willingness to purchase (α = 0.88) and perceived deception (α = 0.90).

To measure the moderating role of image-ingredient use, a new variable was created to classify respondents as either being more *ingredient list*, *image*, or *equally* focused by subtracting a person’s self-reported image-ingredient usage. A negative (positive) score resulting in more ingredient list (image) focused and a zero classifying as equal. 

Regarding the distribution of respondents into the two conditions, they were similarly distributed in terms of gender (*X*^2^(1) = 1.83, *p* = 0.246), age (*F*(1107) = 0.79, *p* = 0.376), *general frequency of fruit juice consumption* (*F*(1107) = 0.44, *p* = 0.507), *liking of 100% fruit juice* (*F*(1107) = 0.07, *p* = 0.800), *liking apple mango fruit juice in general* (*F*(1107) = 3.39, *p* = 0.068), *hedonic score juice in study* (*F*(1107) = 0.63, *p* = 0.430), and “study program” (*F*(1107) = 1.72, *p* = 0.193).

### 4.4. Results

#### 4.4.1. The Effect of Pictorial Packaging Elements on Expected and Perceived Flavor Ratio

Without textual information available, the image on the front of packaging influenced both expected (*t*(106) = −10.98, *p* < 0.001) and perceived flavor ratio (*t*(106) = −3.85, *p* = 0.039). The image depicting more mango led to assimilation towards expected (*M* = −43.4, *SD* = 16.5) and perceived (*M* = −54.4, SD = 17.5) mango flavor. Similarly, with the image depicting more apple, assimilation towards expected (*M* = 14.7, *SD* = 35.2) and perceived (*M* = 18.3, *SD* = 41.3) apple flavor occurred.

#### 4.4.2. The Moderating Effect of Image/Ingredient List Usage on Expected and Perceived Flavor Ratio

To see whether this effect would be stronger for people relying more on *the ingredient list*, *the image* or *equally on both,* people were classified into one of these three categories by subtracting their self-reported scores on image-ingredient list use. No moderating effect was found for expected (*F*(1106) = 1.23, *p* = 0.297) and perceived flavor ratio (*F*(1106) = 0.64, *p* = 0.527), indicating that reliance on the image in creating an expected and perceived flavor ratio did not seem to depend on the self-reported frequency of looking at the image/ingredient list. 

Additionally, scores on the five items for image/label usage between these focus groups were compared (Table 11). As expected, consumers focusing more on the ingredient list showed a trend in higher scores on items 4 and 5 considering the ingredient list with their first purchase. While consumers focusing on the image tended to score higher on the trustworthiness of the image as a content indicator.

#### 4.4.3. Mismatch Perception

A perceived mismatch from expected and perceived flavor intensities was measured (*N* = 108) between the apple and mango conditions. As seen in Table 12, regardless of the image shown on the packaging, the proportion of people reporting a perceived mismatch was the same (*X*^2^(1) = 0.332, *p* = 0.564). 

To check whether a perceived mismatch was caused by a discrepancy between expected and perceived flavor ratios, the additional subtraction scale between an individual’s perceived and expected flavor ratio was looked at. For each person, perceived flavor ratio (−100 to 100) minus expected flavor ratio (−100 to 100) showed that a difference of at least 40 points caused a disconfirmation of expectation, reporting a mismatch. Overall, when disconfirmation was less than 40 points, assimilation towards the depicted image occurred and no mismatch was found. 

#### 4.4.4. Influence of Perceived Mismatch on Perceived Deception and Willingness to Purchase

Similar to the main and second studies, and in line with expectations, a perceived mismatch was found to be a predictor for perceived deception (*F*(1106) = 279.64, *p* < 0.001). In turn, perceived deception was again found to negatively influence intention to purchase (*F*(1106) = 141.32, *p* < 0.001), with an *R*^2^ of 0.571.

## 5. General Discussion

### 5.1. Main Findings

Three studies investigated the effect of ingredient item depiction on expected and perceived flavor ratios. The factual ingredient information was presented in different ways. In the first study, the textual ingredient information was presented next to the packaging, assuming people would be looking at this information. In the second study, this textual information was placed less conspicuously on the bottom of the package, and in the third study, this textual information was not shown at all, assuming that most consumers normally do not make the effort to look at it. From the results, it can be concluded that with a very salient positioning of textual ingredient information, people purely base their expected and perceived flavor ratios on this information. In the next study, textual information still strongly influenced assimilation; however, the ingredient image also influenced expected and perceived flavor ratios. In the third study, with no textual information present at all, the effect of the image on flavor assimilation was very clear. 

#### 5.1.1. Expected and Perceived Flavor Ratios

Combining the three studies, it can be said that the more realistic the setup of the experiment, the more people assimilated their expected and perceived flavor ratio towards the pictorial stimuli on the front of the pack. This is partly in line with findings from previous research on advertisements, suggesting that pictures enhance accessibility of packaging information, attracting more attention and being noticed before verbal information [75]. In this sense, the pictorial information serves as an “advance organizer” as put by Alesandrini [76], creating expectations for interpretation of verbal information. Also, the image on the packaging elicits imagery processing [32], enhancing spontaneous imagination of the product’s taste in representing sensory information from the image in working memory. However, the (non-realistic) salience of the textual information in the stimuli design might have surpassed the vividness of the imagery in the first two studies. As cue utilization theory emphasizes, cue salience is of major importance in creating product expectations and perceptions [77].

From an attention perspective, the vividness of the pictorial stimuli might have gone unnoticed because of the accessibility of the salient textual ingredient information presented next to the packaging. Another explanation of the deviating results might be that respondents in this experiment had unlimited time to extensively evaluate the packaging, while time-pressure and cognitive load are usually common variables during grocery shopping for review, see Reference [68]. In a study by Pieters and Warlop [77], people in a time-pressured condition tended to filter textual information (ingredient information on packaging) more, preferring less cognitively-taxing pictorial image information. 

#### 5.1.2. Mismatch Perception 

Contrasting expectations, it can be said that incongruence of ingredient information expressed in FOP-imagery and ingredient list information (deceptive packaging information) did not lead to mismatch perceptions (hypothesis 3). Despite some findings of incongruence between pictorial and textual packaging elements leading to mismatch perceptions in the first study, this effect was not robust when looking at each level of (in)congruity separately. Only in the deceptive pre-consumption condition depicting more apples on the front of the pack accompanied with an ingredient list showing more mangos, a majority of the respondents perceived this incongruent information as a mismatching. In the other pre-consumption deceptive condition, pictorializing more mango on the front of the pack accompanied with an ingredient list indicating a large amount of apple (which often happens in real-life), the mismatch was not clearly perceived. Perhaps, consumers did not see the first discrepancy as mismatching, because they are used to this way of pictorializing ingredients and actually want to be able to see the “special ingredient” as apple-based juices are more regular compared to the “special” ingredient mango. It might be interesting to further explore this view of the consumer. 

#### 5.1.3. Perceived Deception and Willingness to Purchase

In a combination of the three studies, it can be said that, as predicted, a perceived mismatch between the pictorial and textual packaging elements increased feelings of deception (hypothesis 4), which, in turn, lowered intention to purchase (hypothesis 5). This is in line with the empirical findings of Ozanne and Underwood [53], who found that consumers frequently felt betrayed or duped by different packaging elements, such as unrealistic image size on the packaging and exaggerated nutrition-oriented cues compared to the actual nutritional information. This research confirmed that discrepancy between pictorial and textual information is seen as an intentional form of misleadingness.

Additionally, results showed that a perceived mismatch solely from packaging elements had a smaller effect on perceived deception than a perceived mismatch between expected and perceived flavors. In other words, a consumer feels more betrayed when detecting a mismatch after consuming the product, compared to a mismatch purely from packaging. Perhaps this is because a mismatch from packaging already can be detected before the decision to purchase the product is made, leaving the consumer with the option to opt-out from purchasing, while a mismatch in expected and perceived flavors can only arise after the product is being purchased. 

In addition, this study found that the *perception* of deception was enough to decrease purchase intentions toward the product, whether the ingredient information was *objectively* misleading or not. It would be expected that consumers negatively react to deceptive packaging, in turn lowering intentions to purchase [18]. However, being exposed to a deceptive packaging compared to a non-deceptive packaging did not significantly differ willingness to purchase (although slightly lower) the packaging. From a positive perspective, this indicates that deceptive ingredient information does not convince or persuade consumers to purchase; however, from a critical perspective, the deceptive packaging was virtually as effective in influencing purchase intentions as the non-deceptive packaging. All in all, deceptive packaging will lead to lower intentions to purchase, but only if consumers perceive this deception. Otherwise the packaging appears to be no more or no less favorable than non-deceptive packaging. 

### 5.2. Moderating Role of Cognitive Processing Style and Image-Label Usage

Contrasting expectations, cognitive processing style was not found to be a moderator between the packaging elements of the product and expected flavor ratios, perceived flavor ratios, and mismatch perceptions, whether the ad was objectively misleading or not (hypotheses 6–9). This may be due to the broad focus of the topics of the REI questionnaire. The REI questionnaire measures being a dominant rational or experiential processor in general, while this behavior is very context specific [73]. Added nuance to the questionnaire on self-reported image-ingredient list use (label use) in making food choices was measured in study 3 [74]. It was interesting to see that about 80% reported themselves as “image users”, while in the first two studies we saw that most people relied on textual information. This might be because people were asked explicit questions about the textual information in these studies and again because of the salience of the textual information. Image-label usage also did not moderate the effect of image depiction on flavor assimilation. A more situation-specific measure of rationality and experientiality would be useful [73] and could help interpret these results more carefully.

### 5.3. Contributions and Managerial Implications

A contribution to the literature of this research is the application of an experimental approach to determine whether packaging information is deceptive or not. Until now, most studies have investigated verbal deception in advertisement only [78]. The contribution of this study is to show that deception through graphical elements on packaging is possible as well. More specifically, this study is unique in the marketing domain in the sense that it covered both the pre- and post-consumption evaluation. In other words, both potential misleadingness solely from packaging elements as well as after tasting were investigated, covering *“two moments of truth”* for potential misleadingness. 

Furthermore, insights into the effect of deceptive visualization of ingredients on food product packaging towards purchase intention may be useful for researchers and regulators to develop better knowledge about the conditions under which consumers are most likely to be deceived. This study showed that the less salient (absent) factual ingredient information is presented on the packaging, the more consumers rely on ingredient images to create expected and perceived flavors, that the contrast between visualized ingredients on the FOP label and actual ingredients are not consciously perceived, and how this influences their loyalty to a specific product in terms of willingness to purchase the product. This outcome could add as a guidance for improvement of public policies in order to protect consumers better by either presenting the correct ratio of ingredients on the front of packaging or by making the textual information more salient so the imagery on the front of packs acts as an aesthetic element of the packaging.

### 5.4. Limitations and Future Research

For this research, we chose to use drawn illustrations of the depicted ingredients, while other multiple brands use photographs which might elicit significantly different sensory profiles for the same product [29]. Also, similar results have been found for either using an image of the end product or the ingredient, with depicting the end product resulting in higher ratings of liking and a more positive evaluation of quality attributes [25]. Moreover, the position of the ingredient item list regularly is on the back of the packaging, while in this design it was positioned next to the front of packaging in a single box (first study) or merged on the front of packaging (study 2) or completely absent (study 3). Creating a tangible 3D package design with a realistic ingredient list on the back of the packaging, with different surface/image coverage ratios to examine the effect of (in)congruity between pictorial and textual packaging cues was beyond the scope of this research, but may be interesting for future studies to explore in an even more realistic case study. 

Second, in this study, self-reported image-label usage was used to check the moderating effect of a dominant packaging focus. Additionally, for the 3D packaging, adding an element of monitoring pictorial attention (i.e., eye tracking) could help in understanding and identifying these groups, as consumers tend to overestimate their label use when self-reporting [79]. Also, gaining knowledge in localization of attention could provide ways in which label design could be modified to improve consumers’ ability to locate and effectively utilize factual nutrition information such as the ingredient list [80,81]. 

Third, in the design of the questionnaire, the images of the packaging were no longer available when respondents were asked questions about the packaging. This was done to increase spontaneity and to avoid respondents relying mainly on the ingredient information as shown by the percentages presented with the packaging (studies 1 and 2). However, individual differences in memory capacity, and with this recall of packaging elements, could have moderated the validity and accuracy of the given responses. Fourth, it would be interesting to replicate this study for a more complex product. This study examined a 100% fruit juice, in which solely the ratio of fruit could elicit a perceived mismatch and increase perceived deception. However, many other products containing a more extensive ingredient list such as quark, yoghurt, and cereal (bars) often clearly depict their “special” ingredients (e.g., blueberry) on the front of pack. Investigating to what extent consumers find this way of presenting a product misleading or not could help in creating guidelines for marketing to prevent perceived deception. Moreover, this research could also be extended to the addition of unnatural ingredients and naturalness perception [82].

Finally, it would also be interesting to investigate the effect of the main product title. In this work the title “Apple and Mango” was kept constant across all conditions, but this could have made some respondents more strongly believe that there was more apple than mango. That said, from the results it seems that the dominating source of information was the proportion of ingredients (from studies 1 and 2) and the depicted images (study 3), mostly for the expected flavor ratio.

## 6. Conclusions

The aim of this research was to increase understanding of the effect of depicting ingredient items on the front of packaging on pre- and post-consumption product evaluations in order to create guidelines for pictorial design elements on packaging to protect consumers from potentially being misled. The findings improved our understanding of how pictorials actually used on juice packages in supermarkets affect consumer response. The results showed that consumers did not perceive the incongruity between pictorial and textual information as mismatching. However, a perceived mismatch from packaging, whether objectively deceptive or not, did increase perceived deception, and lower willingness to purchase. This effect was robust for both mismatches, among packaging elements (pre-consumption) and from expected and perceived flavor ratio (post-consumption), but it was more substantial for the post-consumption mismatch. Although the moderating effect of cognitive processing style regarding expected and perceived flavors from pictorial and textual ingredient information was not confirmed, the results indicated that the effect of salient ingredient list information was substantial, independent of a particular processing style or label usage. To the best of our knowledge, this is the first study which has explored the extent to which people rely in packaging images to create flavor expectations with and without information on ingredient proportions and whether tasting of such products overrides this interpretation or is assimilated to any of the two other sources of information. Importantly this study highlights that incongruities between the pictorial and the textual information is generally not perceived as a mismatch by the great majority, but that a mismatch likely leads to perceived deception and lower willingness to purchase. Taken together, these are novel findings that contribute to the literature on consumer psychology, particularly in heuristics and semiotics in food packaging.

## Figures and Tables

**Table 1:** Overview of the 2 (pictorial) × 2 (textual) × 2 (tasting) experimental conditions. A < M refers to either the picture (P) or the text (T), representing less apple than mango; and A > M more apple than mango.

Condition	Pictorial Information	Textual Information	Tasting Involved	(In)Congruity
1	P:A < M	T:A < M	No	Congruent
2	P:A < M	T:A > M	No	Incongruent
3	P:A > M	T:A < M	No	Incongruent
4	P:A > M	T:A > M	No	Congruent
5	P:A < M	T:A < M	Yes	Congruent
6	P:A < M	T:A > M	Yes	Incongruent
7	P:A > M	T:A < M	Yes	Incongruent
8	P:A > M	T:A > M	Yes	Congruent

**Table 2:** Differences across all conditions regarding randomization checks, including a differentiation between the four non-tasting (pre-consumption, PreC) and the four tasting (post-consumption, PostC) conditions.

	Pre-Consumption Conditions					Post-Consumption Conditions					
Randomization Checks	1 Mean (SD)	2 Mean (SD)	3 Mean (SD)	4 Mean (SD)	*p*-Value PreC	5 Mean (SD)	6 Mean (SD)	7 Mean (SD)	8 Mean (SD)	*p*-Value PostC	Global *p*-Value
	*N* = 52	*N* = 53	*N* = 55	*N* = 54	*N* = 214	*N* = 55	*N* = 56	*N* = 55	*N* = 56	*N* = 222	*N* = 436
**Age**	20.86 (2.07)	20.66 (2.14)	21.25 (2.44)	20.43 (2.29)	0.265	21.05 (2.23)	21.04 (2.64)	20.96 (2.17)	20.5 (2.38)	0.550	0.520
**General frequency**	2.69 (0.67)	3.02 (0.87)	2.90 (0.88)	3.09 (0.94)	0.081	3.16 (0.98)	3.00 (0.79)	2.78 (0.76)	2.84 (0.80)	0.075	0.056
**Liking 100% fruit juice**	5.29 (1.65)	5.28 (1.83)	5.15 (1.63)	4.69 (1.89)	0.240	4.87 (1.81)	5.21 (1.70)	5.38 (1.56)	5.55 (1.61)	0.175	0.184
**Liking apple and mango juice**	5.42 (1.72)	5.55 (1.61)	5.38 (1.78)	5.07 (1.74)	0.530	5.35 (1.36)	5.82 (1.52)	5.85 (1.39)	5.5 (1.39)	0.168	0.184
**Attractiveness**	5.46 (1.28)	5.43 (1.32)	5.09 (1.32)	5.09 (1.1)	0.091	5.45 (1.57)	5.61 (1.31)	5.24 (1.47)	5.66 (0.94)	0.346	0.193

Note: Numbers (except age) represent mean scores on each of the scales (seven-point scales for liking 100% fruit juice, liking apple and mango juice, and attractiveness; five-point scale for general frequency).

**Table 3:** ANOVA table with the mean (SD) for each condition on the dependent variables (DVs) including F-values with corresponding significance levels and effect sizes for the main and all possible two- and three-way interaction effects of *pictorial* (P), *textual* (T), and *tasting* (TA). Whenever a respondent had an expected flavor ratio greater (smaller) than zero, this means the participant expected the apple flavor to be more (less) intense compared to the mango flavor of the juice.

DV	Non-Tasting Conditions				Tasting Conditions				Factors in the Model for Each DV						
	P:A < M T:A < M *N* = 52	P:A < M T:A > M *N* = 53	P:A > M T:A < M *N* = 55	P:A > M T:A > M *N* = 54	P:A < M T:A < M *N* = 55	P:A < M T:A > M *N* = 56	P:A > M T:A < M *N* = 55	P:A > M T:A > M *N* = 56	P F-Value (*p*-Value) *ω*^2^	T F-Value (*p*-Value) *ω*^2^	TA F-Value (*p*-Value) *ω*^2^	P × T F-Value (*p*-Value) *ω*^2^	P × Ta F-Value (*p*-Value) *ω*^2^	Ta × T F-Value (*p*-Value) *ω*^2^	P × Ta × T F-Value (*p*-Value) *ω*^2^
Expected Flavor Ratio *	−46.9 ^a^ (33.6)	10.6 ^b^ (42.9)	−46.6 ^c^ (34.5)	20.2 ^d^ (42.9)	-	-	-	-	1.82 (0.178) n.s.	**280.05** **(<0.001)** **0.394**	-	1.58 (0.209) n.s.	-	-	-
Perceived Flavor Ratio	-	-	-	-	−0.7 (47.9)	0.7 (44.8)	−10.2 (50.7)	16.6 (46.0)	0.26 (0.610) n.s.	**4.89 (0.028)** **0.017**	-	**3.98 (0.047)** **0.013**	-	-	-
Perceived Deception	2.5 (1.4)	2.7 (1.6)	3.3 (1.6)	2.6 (1.4)	2.9 (1.5)	2.6 (1.4)	2.7 (1.4)	2.7 (1.5)	1.14 (0.286) n.s.	1.78 (0.182) n.s.	0.25 (0.617) n.s.	1.41 (0.236) n.s.	2.13 (0.145) n.s.	0.17 (0.682) n.s.	3.59 (0.059) n.s.
Purchase Intention	4.5 (1.3)	4.5 (1.1)	3.9 (1.4)	4.4 (1.2)	5.0 (1.3)	4.9 (1.1)	5.1 (1.1)	4.9 (1.3)	1.38 (0.241) n.s.	0.23 (0.634) n.s.	**28.71 (<0.001)** **0.06**	0.17 (0.677) n.s.	2.99 (0.106) n.s.	2.62 (0.106) n.s.	1.15 (0.285) n.s.

Note 1: The significant values in bold are significant at a level of *p* < 0.05. Note 2: P stands for pictorial, T stands for textual, A < M stands for dominant mango, A > M stands for dominant apple. * The total *N* = 436 was taken for expected flavor ratio as no division between tasting and non-tasting conditions could be made at this point. Therefore, *N* for tasting and non-tasting in each combination of pictorial and textual ingredient information was added up forming four conditions, ^a^ *N* = 107, ^b^ *N* = 109, ^c^ *N* = 110, ^d^ *N* = 110. Negative values for expected/perceived flavor ratio indicate a dominant expected/perceived mango flavor, positive scores indicate a dominant expected/perceived apple flavor.

**Table 4:** Count and proportion (%) of perceived mismatch amongst (in)congruent conditions in pre- and post-consumption evaluation.

	Conditions	Perceived Mismatch		Total
		Yes	No	
Pre-consumption *N* = 214	Congruent	17 (16.0%)	89 (84.0%)	106 (100%)
	Incongruent	30 (27.8%)	78 (72.2%)	108 (100%)
Post-consumption *N* = 222	Congruent	33 (29.7%)	78 (70.3%)	111 (100%)
	Incongruent	37 (33.3%)	74 (66.7%)	111 (100%)

**Table 5:** Count and proportion (%) of perceived mismatch amongst (in)congruent conditions in pre-consumption evaluation.

Conditions	Perceived Mismatch		
	Yes	No	Total
Congruent PT:<M	9 (17.3%)	43 (82.7%)	52 (100%)
Congruent PT:A > M	8 (14.7%)	46 (85.2%)	54 (100%)
Incongruent P:A < M T:A > M	7 (13.2%)	46 (86.8%)	53 (100%)
Incongruent P:A > M T:A < M	23 (41.8%)	32 (58.2%)	55 (100%)

Note: P stands for pictorial, T stands for textual, A < M stands for dominant mango, A > M stands for dominant apple.

**Table 6:** Count and proportions (%) of perceived mismatch amongst (in)congruent conditions in pre- and post-consumption evaluation for primary E processors and primary R processors.

			Perceived Mismatch				
Processor groups	Conditions	Pre-Consumption			Post-Consumption		
		Yes	No	Total	Yes	No	Total
Primary E processors *N* = 93	Congruent	11 (22.0%)	39 (78.0%)	50 (100%)	12 (25.0%)	36 (75.0%)	48 (100%)
	Incongruent	13 (30.2%)	50 (69.8%)	43 (100%)	16 (32.7%)	33 (67.3%)	49 (100%)
Primary R processors *N* = 121	Congruent	6 (10.7%)	43 (89.3%)	56 (100%)	21 (33.3%)	42 (66.7%)	63 (100%)
	Incongruent	17 (26.2%)	56 (73.8%)	65 (100%)	42 (33.9%)	83 (66.1%)	125 (100%)

**Table 7:** Demographics and control variables across all conditions.

Randomisation Checks	P and T:A < M Mean (SD) *N* = 54	P:A < M T:A > M Mean (SD) *N* = 54	P:A > M T:A < M Mean (SD) *N* = 54	P and T:A > M Mean (SD) *N* = 54	F-Value *N* = 216	*p*-Value *N* = 216
Males/females	20/34	10/44	14/40	13/41	5.03 *	0.173
E-processor/R-processor	17/37	25/29	24/30	25/29	3.45 *	0.327
Age	22.2 (2.4)	22.6 (4.0)	24.0 (7.4)	22.5 (4.3)	1.51	0.214
General frequency	2.8 (0.9)	2.8 (0.8)	3.0 (0.89)	2.9 (1.0)	0.85	0.417
Liking 100% fruit juice	4.9 (2.1)	4.9 (1.9)	4.7 (1.7)	5.2 (2.0)	0.63	0.598
Liking apple and mango juice	5.5 (1.7)	5.0 (2.0)	5.2 (1.9)	5.2 (2.0)	0.51	0.676
Attractiveness	5.2 (1.3)	4.8 (1.7)	4.8 (1.5)	4.6 (1.6)	1.25	0.291

Note 1: P stands for pictorial, T stands for textual, A < M stands for dominant mango, A > M stands for dominant apple. Note 2: Numbers (except age and Processing Style Influence - PSI score) represent mean scores on each of the scales (seven-point scales for liking 100% fruit juice, liking apple and mango juice, and attractiveness; five-point scale for general frequency). * These values are X^2^ values.

**Table 8:** ANOVA table with mean (SD) for each condition on the dependent variables including F-values with corresponding significance levels for each main and interaction effect of *pictorial* (P) and *textual* (T). Whenever a respondent had an expected flavor ratio greater (smaller) than zero, this means the participant expected the apple flavor of the juice to be more (less) intense compared to the mango flavor of the juice.

Dependent Variable	P:A < M T:A < M *N* = 54	P:A < M T:A > M *N* = 54	P:A > M T:A < M *N* = 54	P:A > M T:A > M *N* = 54	P F (*p*-Value) *ω*^2^	T F (*p*-Value) *ω*^2^	P × T F (*p*-Value) *ω*^2^
Expected Flavor Ratio *	−35.5 (33.7)	−8.9 (44.1)	−29.6 (38.8)	11.4 (44.9)	**5.58 (0.019)** **0.018**	**37.40 (<0.001)** **0.141**	1.68 (0.197) n.s.
Perceived Deception	3.0 (1.6)	3.4 (1.3)	3.1 (1.4)	2.8 (1.4)	1.53 (0.218) n.s.	0.02 (0.887) n.s.	3.26 (0.072) n.s.
Purchase Intention	3.8 (1.5)	3.9 (1.4)	3.9 (1.5)	3.7 (1.2)	0.21 (0.650) n.s.	0.04 (0.846) n.s.	0.34 (0.560) n.s.

Note 1: The significant values in bold are significant at the level *p* < 0.05. Note 2: P stands for pictorial, T stands for textual, A < M stands for dominant mango, A > M stands for dominant apple; * negative (positive) values for expected flavor ratio indicate a dominant expected mango (apple) flavor.

**Table 9:** Count and proportions (%) of perceived mismatch amongst (in)congruent conditions.

Conditions	Perceived Mismatch		
	Yes	No	Total
**Congruent**	25 (23.1%)	83 (76.9%)	108 (100%)
**Incongruent**	26 (24.1%)	82 (75.9%)	108 (100%)

**Table 10:** Count and proportion (%) of perceived mismatch amongst (in)congruent conditions for primary E processors and primary R processors.

Processor Groups	Conditions	Perceived Mismatch		
		Yes	No	Total
Primary E processors *N* = 93	Congruent	8 (19.0%)	34 (81.0%)	42 (100%)
	Incongruent	11 (22.4%)	38 (77.6%)	49 (100%)
Primary R processors *N* = 121	Congruent	17 (25.8%)	49 (74.2%)	66 (100%)
	Incongruent	15 (25.4%)	44 (74.6%)	59 (100%)

**Table 11:** Mean (SD) on a 7 point scale per focus classification group (Image, Ingredient List, and Equal) for each item on image and ingredient list.

Focus Classification	*N*	Image Content Indicator	Image Trustworthy	Image Decorative	Ingredient List- IMPORTANT	Ingredient List-VIEW
Image	88	6.0 (1.9)	6.1 (1.9)	4.5 (2.6)	5.2 (2.5)	5.1 (2.6)
Ingredient List	15	5.7 (2.2)	5.8 (2.3)	5.0 (2.5)	5.8 (1.9)	6.0 (1.6)
Equal	5	5.8 (2.7)	7.2 (0.4)	7.0 (1.0)	5.2 (1.6)	5.4 (2.1)

**Table 12:** Count and proportion (%) of perceived mismatch amongst (in)congruent conditions.

	Perceived Mismatch		
Conditions (*N*)	Yes	No	Total
**P:A < M (54)**	14 (25.9%)	40 (74.1%)	54 (100%)
**P:A > M (54)**	16 (29.6%)	38 (70.4%)	54 (100%)
**Total (108)**	30 (27.8%)	78 (72.2%)	108 (100%)

Note: P stands for pictorial, A < M stands for dominant mango, A > M stands for dominant apple.
